# Preoperative Controlling Nutritional Status Score on Predicting the Postoperative Complications Following Major Hepatopancreatobiliary Surgery

**DOI:** 10.7759/cureus.61349

**Published:** 2024-05-30

**Authors:** Sujan Shrestha, Romi Dahal, Narendra Maharjan, Sumita Pradhan, Bishnu Kandel, Paleswan Joshi Lakhey, Ramesh S Bhandari

**Affiliations:** 1 Colorectal Surgery, Clinic NEO, Kathmandu, NPL; 2 Surgical Gastroenterology, Tribhuvan University Teaching Hospital, Institute of Medicine, Kathmandu, NPL; 3 Surgical Gastroenterology, Pokhara Academy of Health Sciences, Pokhara, NPL; 4 GI, Gastrosurgery, General Surgery, Tribhuvan University Teaching Hospital, Institute of Medicine, Kathmandu, NPL; 5 Surgical Gastroenterology, Maharajgunj Medical Campus, Tribhuvan University Teaching Hospital, Kathmandu, NPL; 6 Surgical Gastroenterology, Tribhuvan University Institute of Medicine, Kathmandu, NPL; 7 Surgical Gastroenterology, Maharajgunj Medical Campus, Tribhuvan University Teaching Hospital, Institute of Medicine, Kathmandu, NPL; 8 Surgery, Tribhuvan University Teaching Hospital, Institute of Medicine, Kathmandu, NPL

**Keywords:** complications, malnutrition, hepatopancreatobiliary surgery, clavien dindo classification, controlling nutritional status score

## Abstract

Introduction

The prognostic significance of the controlling nutritional status (CONUT) score in hepatopancreatobiliary (HPB) surgery has been shown by many studies but the clinical significance of the CONUT score for postoperative short-term outcomes remains controversial. This study aimed to investigate the impact of the CONUT score on early postoperative outcomes in patients following major HPB surgery.

Method

This was a prospective study of 57 patients who underwent major HPB surgery from November 2019 to January 2021 at the Department of Surgical Gastroenterology, Tribhuvan University Teaching Hospital, Nepal.

Result

A total of 57 patients, 25 males and 32 females, were operated on. The number of patients assigned to the normal, mild, and moderate malnutrition groups was 13, 41, and 3, respectively. The high CONUT group (CONUT ³ 2) consisted of 44 patients (77%) and the low CONUT group (CONUT <2) consisted of 13 patients (33%). The overall complications (Clavien-Dindo classification ³1) and major complications (Clavien-Dindo classification ³3) were present in 37 patients (64.9%) and 14 patients (24.6%), respectively. Increased operative time and intraoperative blood loss were associated with an increased incidence of major (OR: 1.01, p: 0.018) and overall (OR: 1.006, p: 0.039) postoperative complications, respectively, in univariate analysis. A high CONUT score was not associated with a higher incidence of overall and major postoperative complications.

Conclusion

In our study, the preoperative CONUT score did not predict the postoperative morbidity following hepatopancreatobiliary surgery.

## Introduction

Surgery is a cornerstone for major hepatopancreatobiliary (HPB) diseases. Hepatic, pancreatic, and biliary surgery has evolved significantly over the past two decades. The incidence of perioperative mortality has decreased to 1-2% following pancreatic resection and 3-4% after major hepatic resections [1-2]. But overall morbidity is still high, 40% following pancreatic resection and 14.5% after major hepatic resections [3-4]. Malnutrition is a common problem among HPB patients. Malnutrition is associated with poor response to therapy as well as poor short-term and long-term outcomes and reduced quality of life. Although several nutritional assessment tools have been developed, the precise evaluation, including nutritional risk and outcome, is still controversial in HPB patients.

The controlling nutritional status (CONUT) score was developed as a nutritional assessment tool by Ignacio et al. in 2005 [5]. It includes evaluation of preoperative serum albumin level, total cholesterol level, and total lymphocyte count. The long-term prognostic significance of CONUT in gastrointestinal cancer has been previously evaluated [6-7]. However, the clinical significance of the CONUT score for postoperative short-term outcomes remains controversial in the field of hepatopancreatobiliary (HPB) surgeries. Rumya et al. (2016) conducted a retrospective study of 417 patients who underwent potentially curative resection for colorectal cancer where they found moderate and severe CONUT scores were associated with higher incidence of overall postoperative (42.4% and 81.8%) and severe complications (27.3% and 63.6%) [8]. Similarly, Yoshida et al. in 2016 performed a retrospective study on 352 patients of esophageal cancer where they found that moderate to severe grade of CONUT was associated with a higher incidence of overall postoperative complication (61.9%) and severe complications (23.8%) [9]. Takagi et al., in their meta-analysis of 10 retrospective studies, concluded that a higher CONUT score was associated with increased risk of early postoperative morbidity and mortality [10-11]. CONUT uses simple parameters and is easy to calculate. Studies on nutritional risk assessment by CONUT and utilization of the risk score in the prediction of early postoperative outcomes are retrospective. Moreover, there is no study from Nepal. Thus, a prospective study is required to evaluate CONUT as a preoperative predictive factor of postoperative complications following major HPB surgery and to counsel the risk of postoperative complications and take preventive measures.

In this regard, we aimed to evaluate the CONUT score in predicting postoperative morbidity and mortality following major HPB surgery.

This article was previously presented as a meeting abstract at the IHPBA World Congress on March 30, 2022.

## Materials and methods

A quantitative prospective observational study was conducted at the Department of Surgical Gastroenterology, Tribhuvan University (TU) Teaching Hospital, Kathmandu, Nepal. Ethical approval was taken from the institutional review committee (Reference number: 227(6-11)990] [E2076/077). All patients who underwent major hepatopancreatobiliary surgery from 26 November 2019 to 15 January 2021 were included in the study. Patients aged less than 16 were not included in this study. Patients who underwent cholecystectomy, common bile duct exploration, and liver hydatid cyst operation were excluded from this study. A convenience sampling method was used. The sample size calculated was 57.

Patients admitted for elective hepatopancreatobiliary surgery within inclusion criteria were included in the study. After consent, all preoperative variables were filled in the prepared proforma. Preoperative variables within one week before operation were only included in this study. The method of assessment of nutritional status according to CONUT is summarized in Table 1.

**Table 1:** Assessment of malnutrition according to CONUT CONUT: controlling nutritional status

Parameter	Undernutrition Degree			
	Normal	Light	Moderate	Severe
Serum Albumin (g/dl) / Score	3.5 – 4.5 / 0	3.0 – 3.49 / 2	2.5 – 2.9 / 4	< 2.5 / 6
Total Lymphocytes/ ml / Score	>1600 / 0	1200 – 1599 / 1	800 – 1199 / 2	<800 / 3
Cholesterol (mg/dl) / Score	>180 / 0	140 – 180 / 1	100 – 139 / 2	<100 / 3
Screening Total Score	0 - 1	2 - 4	5 - 8	9 - 12

Preoperative albumin, total lymphocyte count, and total cholesterol were classified and scored according to their values. The total score of three parameters was categorized as follows: zero to one is normal, two to four is mild malnutrition, five to eight is moderate malnutrition, and nine to twelve is severe malnutrition. Therefore, we decided to take the cut-off value as two and defined low CONUT as within the normal range and high CONUT as two or more in this study. All surgeries were performed by qualified gastrointestinal surgeons. Patients were kept NPO from midnight before surgery. Maintenance IV fluid was started from the night before surgery. Prophylactic antibiotic (e.g., ceftriaxone 1 gm) was given intravenously 30 minutes before incision (at the time of induction of anesthesia). A central venous line (internal jugular) was inserted according to the anesthesiologists’ discretion. Postoperatively, maintenance IV fluid, analgesics (fentanyl, paracetamol, tramadol, ketorolac), PPI (pantoprazole), and antibiotics (piperacillin-tazobactam, ceftriaxone, metronidazole) were prescribed on the cardex as per the surgeon's discretion and changed based on the subsequent course of the patients. The abdominal drain and nasogastric tube were kept as per requirement. All patients were managed in the postoperative ward or ICU for some days as per requirement, were transferred to the ward once oral medications were started, were mobilized, and when they became stable hemodynamically or according to the treating surgeon’s decision. Blood investigations like complete blood count, renal function test, liver function test, blood glucose, and other investigations were done according to the type of surgery or requirement.

Intraoperative variables were noted in proforma only at the end of surgery. Postoperative complications and grading were done using the Clavien-Dindo classification system at the end of 30 days post-surgery during follow-up [12]. We categorized grades I and II as “mild complication” and grades III to V as “severe complication”.

The data of each case was entered in Microsoft Excel for MAC version 16.54 (Microsoft Corporation, Redmond, WA, US) after completion of the proforma at the end of 30 days for each patient. P-value < 0.05 was taken as statistically significant. All data were analyzed using SPSS v23 (IBM Corp., Armonk, NY, US).

## Results

Demographic and clinical characteristics

A total of 57 patients underwent major HPB surgery. The number of patients assigned to the normal, mild, and moderate malnutrition groups was 13, 41, and 3, respectively. There were only three patients with moderate malnutrition and no patients in the severe malnutrition group. Thus, statistical analysis was done by dividing the patients into high and low CONUT groups with a cut-off of two or more defining high CONUT groups or groups with malnutrition. So, according to the CONUT score, we divided 57 patients into 2 groups: 13 (22.8%) were included in the low CONUT group (CONUT <2), and 44 (77.2%) were included in the high COUNT group (CONUT ³2). The mean age of patients was 49.82 ± 15 years, ranging from 21 to 78 years. The clinical characteristics of the patients as per the CONUT score are shown in Table 2.

**Table 2:** Patient characteristics according to the CONUT score # body mass index, ## Eastern Cooperative Oncology Group, ### nutritional risk index, * hypertension, ** diabetes mellitus, CONUT = controlling nutritional status, ASA = American Society of Anesthesiologists

Variables	CONUT <2 (N=13)	CONUT >=2 (N=44)	P-value
Age (years) (Mean±SD)	43.62 ± 12.76	51.66 ± 15.76	0.071
Sex (F/M)	10/3	22/22	0.086
BMI^#^ (kg/m^2^) (Mean±SD)	23.05 ± 3.97	21.77 ± 3.16	0.23
NRI^###^ (Mean±SD)	104.80 ± 10.57	96.50 ± 11.01	0.019
ECOG^##^ 0 - n (%)	12 (92.3%)	32 (72.7%)	0.139
ECOG 1 - n (%)	1 (7.7%)	12 (27.3%)	
ASA 1 - n (%)	3 (23.1%)	28 (63.7%)	0.029
ASA 2 - n (%)	10 (76.9 %)	16 (36.4%)	
Comorbidity			
HTN^*^ - n (%)	5 (62.5%)	9 (52.94%)	0.185
DM^**^- n (%)	3 (37.5%)	8 (42.6%)	0.694
Blood loss (ml) (Mean±SD)	450 ± 306.87	349.77 ± 210.15	0.182
Operative time (minutes) (Mean±SD)	308.46 ± 133.78	294.91 ± 112.40	0.716
Length of stay (days) (Mean±SD)	11.33 ± 3.80	11.96 ± 2	0.918

Almost all clinical variables are statistically equally distributed between high and low CONUT groups except that patients in the high count group had a lower mean nutritional risk index (NRI) (96.50 ± 11.01) and had better American Society of Anesthesiologists (ASA) physical status compared to patients with a low CONUT score.

Perioperative outcomes

**Figure 1:**
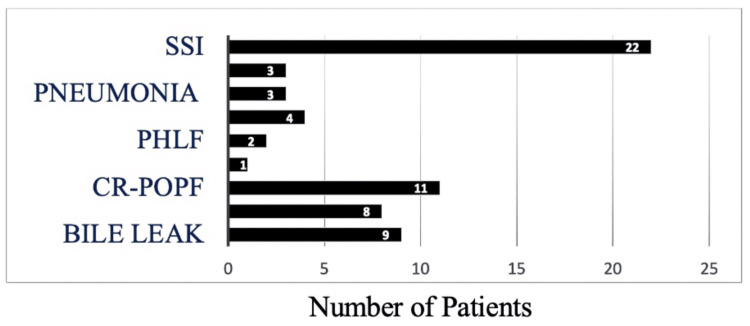
Postoperative complications SSI = surgical site infection, PHLF = post-hepatectomy liver failure, CR-POPF = clinically relevant postoperative pancreatic fistula

Surgical site infection (SSI) was the most common complication in this study. Post-hepatectomy liver failure was present in two patients following liver surgery. Clinically relevant postoperative pancreatic fistula (CR-POPF), bile leak, post-pancreatectomy hemorrhage (PPH), and delayed gastric emptying (DGE) were present in 11, 9, 8, and 1 patients, respectively. Three patients underwent reoperation (Figure 1).

There were 20 patients (35.1%) without any complications. The overall complications (Clavien-Dindo (CD) ³1) and major complications (Clavien-Dindo (CD) ³3) were present in 37 (64.9%) and 14 (24.6%) patients, respectively. There were 5 (8.7%) mortalities (Table 3).

**Table 3:** Perioperative outcomes according to the CONUT score CONUT = controlling nutritional status

Variables	CONUT <2 (n =13)	CONUT >=2 (n =44)	P-value
30-day mortality - n (%)	1 (7.7%)	4 (9.1%)	1
Overall morbidity (CD >= 1) - n (%)	3 (23.1%)	11 (25%)	0.887
Major morbidity (CD >= 3) - n (%)	8 (61.5%)	29 (65.9%)	0.772

Prediction of major postoperative complications

Univariate Analysis

Patients with no major postoperative complications had good performance status compared to patients with major postoperative complications. The mean operative time and intraoperative blood loss were 387.86 ± 107.57 and 517.86 ± 259.15, respectively, in patients with major postoperative complications, which were statistically significant compared to patients without major postoperative complications. The CONUT score was similar between both groups (Table 4).

**Table 4:** Predictors of major postoperative complications BMI = body mass index, ECOG = Eastern Cooperative Oncology Group, NRI = nutritional risk index, CONUT = controlling nutritional status, ECOG = Eastern Cooperative Oncology Group

Variables	Postoperative Complications (CD<= 3)	Postoperative Complications (CD<3)	P- Value
Sex F – n (%)	6 (42.9%)	26 (60.5%)	0.249
Sex M – n (%)	8 (57.1%)	17 (39.5%)	
Age (years) (Mean±SD)	55.57 ± 17.12	47 ± 14.52	0.15
BMI (Mean±SD)	21.81 ± 3.69	22.14 ± 3.30	0.748
NRI (Mean±SD)	95.34 ± 12.55	99.39 ± 10.93	0.251
Intraoperative blood loss (ml) (Mean±SD)	517.86 ± 259.15	325 ± 210.75	0.007
Operative time (minutes) (Mean±SD)	387.86 ± 107.57	268.74 ± 104.60	0.001
ECOG 0 – n (%)	7 (50%)	37 (86%)	0.005
ECOG 1 – n (%)	7 (50%)	6 (14%)	
CONUT <2 – n (%)	3 (21.4%)	10 (23.3%)	0.887
CONUT >=2 – n (%)	11 (78.6%)	33 (76.7%)	

Multivariate Analysis

ECOG 1, increased intraoperative blood loss, and operative time predicted major postoperative complications in univariate analysis. In multivariate analysis, the patient's ECOG 1 and increased intraoperative time were predictive of major postoperative complications following major HPB surgery (Table 5).

**Table 5:** Predictors of major postoperative complications (multivariate analysis) BMI = body mass index, ECOG = Eastern Cooperative Oncology Group, OT = operative time, NRI = nutritional risk index

Variables	Multivariate analysis			
	OR	95% CI		P-value
		lower	upper	
Age	0.985	0.925	1.050	0.646
Sex(male)	3.567	0.328	38.730	0.296
ECOG 1	7.712	1.057	48.687	0.044
BMI	1.091	0.773	1.539	0.622
NRI	0.959	0.860	1.068	0.446
OT time	1.010	1.000	1.019	0.043
Blood loss	1.004	0.998	1.009	0.186
CONUT>=2	2.361	0.136	40.977	0.555

## Discussion

Postoperative outcomes following major surgery depend upon various preoperative, intraoperative, and postoperative variables. The preoperative nutritional status of the patient is one of the important preoperative factors that determine the postoperative outcomes. The association between preoperative malnutrition and poor postoperative outcomes has been shown by many studies not only in gastrointestinal but also in other major surgeries [13-15]. There are many tools utilized in the literature to assess the nutritional status of surgical patients [16]. Controlling nutritional status (CONUT) is a nutritional assessment tool introduced by de Ulıbarri et al. in 2005 [5]. In this study, we have used CONUT to assess the nutritional status of our patients.

We divided our patients into two groups: patients with a CONUT score of less than two as the low CONUT group and those with a CONUT score of CONUT>=2 as the high CONUT group. The cutoff of CONUT score two to divide patients into two groups is based on the CONUT original study and other similar studies [5-6]. So, as per the CONUT score, patients with low CONUT (CONUT <2) have normal nutrition, and patients with high CONUT (CONUT>=2) have some form of malnutrition. The high CONUT group can be further categorized into light, moderate, and severe malnutrition [5]. In this study, we have not further categorized the high CONUT group. According to the CONUT score, we divided 57 patients into 2 groups: 13 (22.8%) were included in the low CONUT group (CONUT <2), and 44 (77.2%) were included in the high COUNT group (CONUT>=2). The mean age of patients in the high CONUT group was 51.66 ± 15.76, which did not differ from patients in the low CONUT group. The high CONUT group had lower BMI compared to low CONUT group patients but this was not statistically significant.

The prevalence of malnutrition in patients undergoing HPB surgery varies in the available literature. Most studies include either pancreatic, hepatic, or biliary surgery separately. The incidence of malnutrition as per CONUT ranges from 9.1% to 43.7% [17]. As discussed earlier, the wide difference in the prevalence of malnutrition in HPB patients is due to two main variability. First, most of the studies include either pancreatic, hepatic, or biliary surgery separately. There is a lack of studies incorporating combined HPB surgery. Second are the different cutoff values used to define malnutrition as per CONUT. In this study, the prevalence of malnutrition is 77.2% (n=44) when a cutoff of two or more was used to define the malnutrition group as per CONUT.

The CONUT score was found to be an independent predictor of survival following curative resection of colorectal, esophageal, and other hepatobiliary cancers. There are mixed results on the association between CONUT score and postoperative complications. Nayoa Yoshida et al. concluded that moderate to severe malnutrition as per CONUT was associated with a high risk for postoperative complications following esophagectomy for esophageal carcinoma [9]. In our study, the preoperative CONUT score did not predict the overall and major postoperative complications following HPB surgery. In this study, there were more patients with mild malnutrition and only three patients with moderate malnutrition. In other words, mild malnutrition has no association with postoperative complications. The association of moderate to severe malnutrition with postoperative complications requires further studies incorporating more cases of moderate to severe malnutrition. However, in clinical scenarios, patients with moderate to severe malnutrition might be not fit for major HPB surgeries. And, these severely malnourished groups might go for prehabilitation and then major surgery only after improvement in their nutritional status.

All preoperative variables like age, gender, BMI, and NRI were equally distributed between both groups. There were more patients with ASA 2 in the low CONUT group which was statistically significant (p=0.029). Other variables like intraoperative blood loss, total operative time, and length of stay were similar between both CONUT groups. The association between operative time and the risk of postoperative complications is shown by many studies. Hang Cheng et al., in their systemic review and meta-analysis concluded that prolonged operative time is associated with an increased risk of postoperative complications [18]. Takagi et al., in their retrospective study of 721 patients undergoing major HPB surgery, demonstrated that the operative time (OR 1.79; p = 0.005) and blood loss (OR 1.66; p = 0.03) were identified as independent risk factors for serious postoperative morbidity [10]. In this study, we found ECOG (OR 7.712; p = 0.044) and operative time (OR 1.010; p = 0.043) as independent risk factors for major postoperative complications. These results supported previous findings that operative time was an independent predictor of adverse early outcomes following HPB surgery [19]. The reasons that serious postoperative morbidities only depended on intraoperative factors rather than preoperative factors might be explained by the more invasive nature of the surgery. Accordingly, decreasing operative time may decrease the risk of postoperative morbidities after HPB surgery.

Limitations of the study

Despite our important findings from this prospective study, this study has a few limitations. The heterogeneity of the study population may be the first limitation. Around 56 % of patients were operated on for pancreas-related pathology and the rest of the patients were divided among hepatic and biliary surgery. Both benign and malignant pathologies were included in the study. The severity of malnutrition is associated with an increased risk of postoperative complications [20]. The inclusion of all malnourished patients into a single group might be the other limitation. We have classified our study cohort into two groups, one without malnutrition and one with malnutrition because we had no patients and only three patients with severe and moderate malnutrition according to CONUT scores, respectively.

## Conclusions

Malnutrition is a common problem in patients with a major hepatopancreatobiliary disease, more than two-thirds of our HPB patients were malnourished as per the CONUT score. CONUT is a simple and convenient tool to assess nutritional status before major HPB surgery. In our study, the CONUT score did not predict the risk of postoperative complications following major HPB surgery. Therefore, the CONUT score has a certain value in the preoperative nutritional assessment of hepatopancreatobiliary patients but its use to predict postoperative complications following major HPB surgery mandates further studies.
